# Small ruminant pasteurellosis in Tigray region, Ethiopia: marked serotype diversity may affect vaccine efficacy

**DOI:** 10.1017/S095026881600337X

**Published:** 2017-01-23

**Authors:** K. BERHE, G. WELDESELASSIE, J. BETTRIDGE, R. M. CHRISTLEY, R. D. ABDI

**Affiliations:** 1 Addis Ababa University, College of Veterinary Medicine and Agriculture, Department of Clinical Studies, Bishoftu, Oromia, Ethiopia; 2 Mekelle Regional Veterinary Diagnostic and Research Laboratory, Mekelle, Tigray, Ethiopia; 3 Institute of Infection and Global Health, University of Liverpool, Leahurst Campus, Liverpool, UK; 4 International Livestock Research Institute, Nairobi, Kenya; 5 The University of Tennessee, Department of Animal Science, Knoxville, TN, USA

**Keywords:** Co-infection, *B. trehalosi*, *M. haemolytica*, *P. multocida*, serotype diversity

## Abstract

The aim of this study was to investigate the prevalent *Bibersteinia, Mannheimia* and *Pasteurella* serotypes, risk factors and degree of serotype co-infections in sheep and goats in the Tigray region of Ethiopia. Serum was collected from 384 sheep and goats from the Tanqua-Abergelle district of Tigray region using cross-sectional random sampling. An indirect haemagglutination test was used for serotyping. Risk factors for infections were evaluated by logistic regression. Potential clustering of multiple serotypes within individual animals due to common risk factors was evaluated by redundancy analysis. Eight serotypes were identified: all studied animals were serologically positive for at least one serotype. Overall, 355 (92·45%) of the animals were infected by four or more serotypes. Of the five risk factors studied, peasant association (PA), animal species, age (serotype A1), and bodyweight (serotype T15) were significantly associated with infection, but sex was not significant. Only PA explained a significant proportion of the variation (adjusted *R*^2^ = 0·16) in the serological responses. After the effect of PA was accounted for, T3 and T4; A7 and *Pasteurella multocida* A; and A7 and T10 were positively correlated for co-infection, while T4 and T10 were less likely to be found within the same animal. Diverse serotypes were circulating in the Tigray region and could be a challenge in selecting serotypes for vaccine.

## INTRODUCTION

*Mannheimia haemolytica* and *Pasteurella multocida* have been identified as major economic problems in ruminant production worldwide. *P. multocida* has 16 serotypes using lipopolysaccharide antigens as tested by a gel diffusion precipitation test [1] although it has five serogroups (A, B, D, E, F) using capsular antigens as tested by a passive haemagglutination test [2]. *M. haemolytica* is composed of a collection of 17 serotypes using capsular antigens as assayed by indirect haemagglutination [3] or co-agglutination test [4]. Globally, the 17 *M. haemolytica* serotypes were reorganized into *Bibersteinia trehalosi* containing four serotypes (T3, T4, T10, T15), *M. haemolytica* containing 12 serotypes (A1, A2, A5–A9, A12–A14, A16, A17) and *M. glucosida* with one serotype (A11) [5]. Despite the 17 serotypes, additional untypable isolates are recognized which account for more than 10–15% of the clinical isolates from ruminants [6].

Serotypes have demonstrated varying degrees of pathogenicity and virulence as assayed by clinical illness, post-mortem lung lesions and bacterial re-isolation rate [7]. Immunity is serotype specific and few [8] or no [4] serotypes cross-react. Distinct serotype associations with specific host species are noted and some serotypes have also distinct disease syndromes. Thus, *P. multocida* serotypes B and E cause haemorrhagic septicaemia of cattle, buffalo, goat, camel and deer; serotype D causes atrophic rhinitis of pigs and rabbits; serotypes A and D cause enzootic pneumonia and shipping fever of cattle, sheep and pigs; and serotypes A and F cause avian cholera of all bird species [9]. Similarly, *M. haemolytica* serotype A1 causes pneumonic pasteurellosis in cattle; *M. haemolytica* serotype A2 causes pneumonic pasteurellosis in sheep and goats and *B. trehalosi* serotypes cause septicaemic pasteurellosis in sheep and goats. Interspecies transmission of serotypes among domestic ruminants, as well as between domestic and wild ruminants, has been reported [10, 11], although some reports suggest that this is a rare epidemiological event [6].

Pasteurellosis and mannheimiosis are mainly influenced by a wide variety of environmental and management risk factors. Thus, the reduction or even elimination of such predisposing factors is of major importance. Antimicrobial drugs represent the most powerful tools to control such infections. However, increasing rates of antimicrobial resistance may markedly reduce the efficacy of the antimicrobial agents used to control *Pasteurella* and *Mannheimia* infections [12]. Vaccination is the best alternative practical control strategy to reduce the incidence and burden of the disease and to minimize antimicrobial use. Currently, several vaccine types exist against pasteurellosis globally [13]. Problems with vaccination arise where there is more than one serotype circulating, due to the lack of cross-protection [8].

At present, only a monovalent *P. multocida* serotype A vaccine is commercially available in Ethiopia [14], although *P. multocida* serotypes A and D [15] and 11 serotypes belonging to *M. haemolytica* [16] have long been detected. Consequently, repeated outbreaks are reported in Ethiopia [15, 17–19] even among vaccinated sheep and goats, which practitioners and communities ascribe to vaccine failure.

Serotypes have different levels of virulence, host-species adaptability with possible inter-species transmissibility, antigenicity, immunogenicity, drug resistance and a lack of inter-serotype cross-reactivity [4, 7]. In Ethiopia, however, detailed information is lacking. Routine serotyping is not practised in Ethiopia due to the time and expense involved, and the lack of commercially available antisera, so that the serotypes of the circulating and outbreak-causing isolates remain unknown. Thus, the antigenicity, immunogenicity and cross-reactivity among the serotypes of the different eco-zones have not been studied. Therefore, this study was conducted to evaluate the (i) abundance, distribution and risk factors of infection for the different serotypes, and (ii) serotype mixed co-infections with potential clustering of *Pasteurella, Mannheimia* and *Bibersteinia* in sheep and goats in some selected areas of Tigray region.

## MATERIALS AND METHODS

### Study area

This study was conducted in five peasant associations (PAs), namely, Lemlem, Negede-Brhan, Gruwure, Mearey and Emba-Rufael of Tanqua-Abergelle district in Tigray regional state. A PA is the lowest administrative structure averaging about 50–80 km^2^ of land, with multiple PAs in each district. The study district is located 863 km north of Addis Ababa at 13^o^ 14′ 06″ N latitude, 38^o^ 58′ 50″ E longitude. It has a lowland agro-ecology with elevation between 938 and 2202 m a.s.l., daily maximum temperature range between 21 °C and 31 °C and annual rainfall between 400 and 650 mm, the rainfall patterns are characterized as low and erratic. The production system in the district is extensive with traditional housing and grazing of natural pasture system. Mixed livestock farming is practised comprising of cattle, sheep, goats, mules, horses, and donkeys. Crops are grown mainly for their grains and to make use of crop residues for animal feed [20].

### Study animals, design and sample size

Tanqua-Abergelle district was selected purposively due to its large population of sheep and goats, high reports of respiratory disease and pasteurellosis challenge and being the source of small ruminants for slaughter at a number of abattoirs in Tigray region, including the Abergelle export slaughterhouse of Mekelle. Abergelle sheep and goats, which were the focus of this study, have previously been characterized by household ownership, management practices, feed resource base, (re)productive performances, consumption and marketing [21]. Abergelle goats are a meat-type breed [22] although they can also produce milk [23].

A cross-sectional study design was employed. To the best of our knowledge, there have been no previous studies on pasteurellosis in the district. Thus, the sample size of study animals was calculated according to Thrusfield [24] using a 50% expected prevalence and a 95% confidence interval (*Z* = 1·96) with a 5% desired absolute precision. The calculated sample size was 384 using the formula: *n* = 1·96^2^*(0·5)*(1–0·5)/0·0025. Of the 23 PAs in the district, five PAs were selected randomly. Farmers who had sheep and goats were selected from registers of PA members using simple random sampling and all animals belonging to selected farmers were included in the study. Selection of additional participants continued until the calculated number of ruminants was achieved. The name of the PA and the owner of the small ruminants, as well as species, age, sex and bodyweight of each study animal were recorded during the survey.

### Sample collection, transportation and laboratory analysis

From each animal 10 ml blood was collected aseptically from the jugular vein into a plain tube. The blood was allowed to clot without shaking for 2 h at room temperature, and stored horizontally overnight at 4 °C. The serum was then separated from the clot by centrifugation at 3000 rpm for 10 min and transferred to cryovials, labelled and transported in an icebox to the district veterinary clinic and kept frozen (–20 °C) until analysis. The collected sera were transported on ice to the National Veterinary Institute, Bishoftu, where they were tested using an indirect haemagglutination (IHA) test as described by Biberstein *et al*. [3] and Biberstein & Thompson [25]. An agglutination rate of >50% was taken as positive [26]. Positive agglutination was confirmed by the presence of a dense clot at the bottom of the microtitre well and negative result by the presence of uniform suspension of the red blood cells. Each sample was tested for eight different serotypes, namely, *M. haemolytica* types A1, A2, A7, *P. multocida* type A and *B. trehalosi* types T3, T4, T10 and T15. Reference serotypes were obtained from CIRAD-EMVT, France.

### Data analysis

Microsoft Excel 2007 (Microsoft Corp., USA) was used for data management. Descriptive statistics were used to summarize the data. PA, age, sex, species and bodyweight of the animals were considered as potential risk factors for *Pasteurella, Mannheimia* and *Bibersteinia* seropositivity. Associations with the dependent variable (seropositivity) were assessed initially using cross-tabulation and univariable logistic regression using SPSS software v. 20 (IBM Corp., USA) to compute the odds ratio associated with potential risk factors. Non-collinear variables that presented a *P* value ⩽0·25 in the univariable analyses were included in the multivariable logistic regression model. In the final model, results were considered significant at *P* < 0·05.

### Multivariable analysis

To identify clustering of seropositivity to multiple serotypes within individual animals it was necessary to allow for potential clustering of seropositivity due to common risk factors, as some PAs, and also the species of the animals were risk factors for more than one serotype. We used redundancy analysis (RDA), a form of multivariable analysis that combines a principal component analysis, to identify clusters, with regression to identify significant explanatory variables, in order to address this question. RDA was performed using R software v. 3·0·2 (R Core Team 2013), using the R package ‘vegan’ [27], according to the methods described by Borcard *et al.* [28]. The binary data for each of the eight *Mannheimia, Pasteurella* and *Bibersteinia* strains was used to construct a distance matrix of responses, which was transformed using a Hellinger transformation, to reduce the impact of double negatives in the dataset, as described by Borcard *et al*. [28]. The categorical explanatory variables considered were PA, age, sex, species and bodyweight. A forward selection process was used to select significant variables which explained the greatest proportion of variance in the response data, and permutation tests used to test significance of RDA axes. Triplots were produced according to correlations between variables (scaling 2 in the vegan package).

## RESULTS

### Univariable analysis of serotype distribution in sheep and goats in different PAs

Of 384 sheep and goats studied, 98·7%, 66·9% and 98·7% were seropositive to *M. haemolytica, P. multocida* serotype A and *B. trehalosi*, respectively. In total, eight serotypes were detected in the study area. Higher prevalence of serotypes A1, A2, T3, T4 and T15 were observed in goats than in sheep; this association was statistically significant (*P* < 0·05) for all serotypes except T15. Serotypes A7 and T10 were more prevalent in sheep, as was *P. multocida* serotype A, overall, although this latter finding was not statistically significant (Table 1). For two serotypes (A1 and T15) prevalence did not differ significantly between the five PAs, although all other serotypes showed statistically significant (*P* < 0·05) variation compared to the reference PA (Lemlem). In Mearey, Negede-Brhan and Emba-Rufael, A2, T3 and T4 prevalence was lower; while prevalence of A7, *M. haemolytica* and T10 (except in Emba-Rufael) were significantly higher, compared to Lemlem. In Gruwure, only T4 prevalence was significantly lower, while A7 and *M. haemolytica* prevalence were higher (Table 1).

**Table 1.** *M. haemolytica*, *P. multocida* and *B. trehalosi* serotype prevalence and the associated risk factors in sheep and goats in different peasant associations of Tigray region

Variables	Summary statistics	A1	A2	A7	Total A	MA	T3	T4	T10	T15	Total T
Sheep (*n* = 192)	Positive (%)	144 (75·0)	132 (68·8)	139 (72·8)	187 (97·4)	134 (69·8)	149 (77·6)	99 (51·6)	90 (46·9)	167 (87·0)	189 (98·4)
Goats (*n* = 192)	Positive (%)	161 (83·9)	151 (78·6)	112 (58·3)	189 (98·4)	123 (64·1)	175 (91·8)	141 (73·4)	69 (35·9)	177 (92·2)	190 (99·0)
Total (*n* = 384)	Positive (%)	305 (79·4)	283 (73·7)	251 (65·5)	379 (98·7)	257 (66·9)	324 (84·4)	240 (62·5)	159 (41·4)	344 (89·6)	379 (98·7)
Sheep		Ref.	Ref.	Ref.	Ref.	Ref.	Ref.	Ref.	Ref.	Ref.	Ref.
Goats	OR (95% CI)	1·73 (1·04–2·86)	1·67 (1·05–2·65)	0·52 (0·34- 0·80)	1·67 (1·05–2·65)	0·77 (0·50–1·18)	2·97 (1·63–5·43)	2·59 (1·69–3·98)	0·64 (0·42–0·96)	1·77 (0·90–3·46)	1·51 (0·25–9·12)
	*P* value	0·033	0·0284	0·0031	0·0284	0·2333	0·0004	<0·001	0·03	0·098	0·6548
Lemlem (*n* = 80)	Positive (%)	63 (78·8)	65 (81·3)	80 (10·1)	75 (93·8)	25 (31·3)	78 (97·5)	67 (83·8)	11 (13·8)	71 (88·8)	80 (100·0)
Negede-Brhan (*n* = 80)	Positive (%)	62 (77·5)	54 (67·5)	64 (80·0)	79 (98·8)	71 (88·8)	60 (75·0)	47 (58·8)	55 (68·8)	76 (95·0)	79 (98·8)
Gruwure (*n* = 82)	Positive (%)	67 (81·7)	75 (91·5)	62 (75·6)	81 (98·8)	60 (73·2)	75 (91·5)	57 (69·5)	19 (23·2)	77 (93·9)	81 (98·8)
Mearey (*n* = 76)	Positive (%)	61 (80·3)	50 (65·8)	63 (82·9)	76 (100·0)	57 (75·0)	56 (73·7)	22 (28·9)	56 (73·7)	59 (77·6)	73 (96·1)
Emba-Rufael (*n* = 66)	Positive (%)	52 (78·8)	39 (59·1)	54 (81·8)	65 (98·5)	44 (66·7)	55 (83·3)	47 (71·2)	18 (27·0)	61 (92·4)	66 (100·0)
Lemlem		Ref.	Ref.	Ref.		Ref.	Ref.	Ref.	Ref.	Ref.	
Negede-Brhan*	OR (95% CI)	0·92 (0·43–1·96)	0·47 (0·23–0·99)	35·5 (14·24–88·48)	—	17·35 (7·49–40·16)	0·07 (0·01- 0·34)	0·27 (0·13–0·58)	13·8 (6·24- 0·48)	2·40 (0·71–8·16)	—
	*P* value	0·8484	0·0486	<0·001	—	<0·001	<0·001	<0·001	<0·001	0·1584	—
Gruwure*	OR (95% CI)	1·2 (0·55–·61)	2·47 (0·9–6·43)	27·51 11·32–66·84)	—	6·00 (3·04–11·84)	0·27 (0·05–1·36)	0·44 (0·20–0·94)	1·89 (0·83–4·28)	1·95 (0·62–6·10)	—
	*P* value	0·63	0·0635	<0·001	—	<0·001	0·1141	0·0348	0·1263	0·2500	—
Mearey*	OR (95% CI)	1·09 (0·50–2·39)	0·44 (0·21- 0·92)	43·00 (16·74–110·50)	—	6·60 (3·27–13·32)	0·07 (0·01–0·31)	0·07 (0·036–0·17)	17·6 (7·76–39·7)	0·43 (0·18–0·06)	—
	*P* value	0·8151	0·0302	<0·001	—	<0·001	<0·001	<0·001	<0·001	0·0670	—
Emba-Rufael*	OR (95% CI)	1·00 (0·45–2·22)	0·33 (0·15–0·70)	39·93 (15·26–104·49)	—	4·40 (2·19–8·83)	0·12 (0·02–0·60)	0·48 (0·21–1·06)	2·35 (1·02–5·42)	1·54 (0·49–4·86)	—
	*P* value	0·9956	0·0039	<0·001	—	<0·001	0·0092	0·0714	0·0448	0·4557	—

A, *Mannheimia haemolytica* serotype A; MA, *Pasteurella multocida* serotype A; T, *Bibersteinia trehalosi* serotype T; OR, odds ratio, CI, confidence interval; Ref., reference category.

* Univariable analysis where sheep and Lemlem peasant association were references.

### Univariable analysis of serotype distribution in small ruminants of different age, bodyweight and sex

Age of the animals was not associated with seven of the serotypes studied, the exception was *M. haemolytica* serotype A1 which was significantly more prevalent (*P* < 0·05) in age groups >2·5 years compared to the reference age group (<1 year). Only *B. trehalosi* T15 showed a statistically significant association with bodyweight, being more prevalent in animals weighing 11–20 kg compared to the reference bodyweight group (1–10 kg) (*P* < 0·05). Sex of the animals did not have any significant (*P* > 0·05) association with any of the eight serotypes studied (Table 2).

**Table 2.** *M. haemolytica*, *P. multocida* and *B. trehalosi* serotype prevalence in different age, bodyweight and sex as risk factors in sheep and goats in some locations of Tigray region

	Summary statistics	A1	A2	A7	Total A	MA	T3	T4	T10	T15
**Age**										
<1 yr (*n* = 19)	Positive (%)	10 (66·7)	11 (73·3)	7 (46·7)		7 (46·0)	15 (78·9)	11 (73·8)	5 (33·3)	11 (73·3)
1–2½ yr (*n* = 113)	Positive (%)	70 (77·8)	67 (74·4)	56 (62·2)		62 (68·9)	100 (88·5)	64 (71·1)	38 (42·2)	83 (92·2)
>2½ yr (*n* = 252)	Positive (%)	225 (80·6)	205 (73·5)	188 (67·6)		188 (67·4)	209 (82·9)	165 (59·1)	116 (41·6)	250 (89·6)
<1 yr	Ref.	Ref.	Ref.	Ref.		Ref.	Ref.	Ref.	Ref.	Ref.
1–2½ yr	OR (95% CI)	2·69 (0·97–7·45)	0·76 (0·25–2·28)	0·90 (0·30–2·71)		1·62 (0·56–4·38)	2·05 (0·59–7·12)	0·78 (0·28–2·15)	1·51 (0·55–4·12)	1·88 (0·54–6·49)
	*P* value	0·0557	0·6298	0·8573		0·3411	0·2580	0·6453	0·4173	0·3148
>2½ yr	OR (95% CI)	3·17 (1·20- 8·32)	1·14 (0·39- 3·30)	0·58 (0·20–1·66)		1·45 (0·56–3·75)	1·29 (0·41–4·09)	1·06 (0·40–2·81)	1·10 (0·42–2·91)	2·78 (0·85–9·13)
	*P* value	0·0190	0·8052	0·3108		0·4384	0·6586	0·8934	0·8334	0·0903
**Weight**										
<0 kg (*n* = 15)	Positive (%)	10 (66·7)	11 (73·3)	7 (46·7)		7 (46·0)	15 (78·9)	11 (73·8)	5 (33·3)	11 (73·3)
11–20 kg (*n* = 90)	Positive (%)	70 (77·8)	67 (74·4)	56 (62·2)		62 (68·9)	100 (88·5)	64 (71·1)	38 (42·2)	83 (92·2)
>20 kg (*n* = 279)	Positive (%)	225 (80·6)	205 (73·5)	188 (67·6)		188 (67·4)	209 (82·9)	165 (59·1)	116 (41·6)	250 (89·6)
<10 kg	Ref.	Ref.	Ref.	Ref.		Ref.	Ref.	Ref.	Ref.	Ref.
										
11–20 kg	OR (95% CI)	1·75 (0·53–5·71)	1·05 (0·30–3·65)	1·88 (0·62–6·71)		2·53 (0·83–6·71)	2·25 (0·53–9·50)	0·89 (0·26–3·06)	1·46 (0·47–4·27)	4·31 (1·08–17·13)
	*P* value	0·3537	0·9274	0·2598		0·1006	0·2698	0·8600	0·5186	0·0379
>20 kg	OR (95% CI)	2·08 (0·68- 6·34)	1·01 (0·31–3·26)	2·36 (0·83–6·71)		2·36 (0·83–6·71)	1·20 (0·32–4·42)	0·52 (0·16–1·69)	1·42 (0·47–4·27)	3·13 (0·93–10·48)
	*P* value	0·3537	0·9902	0·1070		0·1070	0·7809	0·2818	0·5292	0·0636
**Sex**										
Male (*n* = 77)	Positive (%)	62 (80·5)	50 (64·9)	53 (68·8)	76(98·7)	55 (71·4)	66 (85·7)	45 (58·4)	30 (39·0)	69 (89·6)
Female (*n* = 307)	Positive (%)	243 (79·2)	233 (75·9)	198 (64·7)	300 (97·7)	202 (65·8)	258 (84·0)	195 (63·5)	129 (42·0)	275 (89·6)
Male	Ref.	Ref.	Ref.	Ref.	Ref.	Ref.	Ref.	Ref.	Ref.	Ref.
										
Female	OR (95% CI)	0·91 (0·49–1·72)	1·70 (0·99–2·90)	0·83 (0·48–1·41)	0·56 (0·06–4·63)	0·76 (0·44–1·33)	0·87 (0·43–1·78)	1·23 (0·74–2·06)	1·13 (0·68–1·89)	0·99 (0·43–2·25)
	*P* value	0·7909	0·0523	0·4963	0·594	0·3486	0·7175	0·4112	0·6262	0·9931

A, *Mannheimia haemolytica* serotype A; MA, *Pasteurella multocida* serotype A; T, *Bibersteinia trehalosi* serotype T; OR, odds ratio, CI, confidence interval; Ref., reference category.

### Multivariable logistic regression for evaluation of risk factors in the final model

A multivariable logistic regression identified that only PA (location) and species were significant risk factors for the majority of the serotypes. *M. haemolytica* (serotypes A2 and A7), and *B. trehalosi* (serotypes T3, T4 and T10) infections were significantly associated with particular PAs. However, some serotypes and PAs had no association: *M. haemolytica* serotype A2 *vs*. Negede-Brhan; *B. trehalosi* serotypes T3, T4 and T10 *vs*. Gruwure; and *B. trehalosi* serotype T10 *vs*. Negede-Brhan. After controlling for PA, goats had increased odds of *B. trehalosi* serotype T3 and T4 seropositivity (Table 3).

**Table 3.** *M. haemolytica*, *P. multocida* and *B. trehalosi* serotypes identified in goats in reference to sheep and in different peasant associations in reference to Lemlem in some locations of Tigray region

Serotype	Summary statistics	Species		Peasant associations				
		Sheep (*n* = 192)	Goats (*n* = 192)	Lemlem (*n* = 80)	Negede-Brhan (*n* = 80)	Gruwure (*n* = 82)	Mearey (*n* = 76)	Emba-Rufael (*n* = 66)
A1	OR (95% CI)	Ref.	1·85 (1·1–3·1)	1	1·02 (0·47–2·18)	1·36 (0·61–2·99)	1·42 (0·63–3·21)	1·18 (0·52–2·67)
	*P* value		3·135			0·441	0·394	0·688
A2	OR (95% CI)	Ref.	1·50 (0·91–2·46)	1	0·50 (0·24 −1·05)	2·68 (1·02–7·04)	0·52 (0·24–1·12)	0·368 (0·17–0·78)
	*P* value		0·107		0·07	0·044	0·097	0·009
A7	OR (95% CI)	1	0·87 (0·51–1·50)	1	35·40 (14·18–88·38)	27·29 (11·19–66·54)	41·39 (15·75–108·76)	39·26 (14·90–103·46)
	*P* value		0·639		0·00	0·00	0·00	0·00
MA	OR (95% CI)	1	1·04 (0·63–1·69)	1	17·46 (7·51–40·56)	6·04 (3·04–12·01)	6·71 (3·22–13·97)	4·44 (2·18–9·03)
	*P* value		0·87		0·00	0·00	0·00	0·00
T3	OR (95% CI)	1	2·46 (1·30–4·64)	1	0·08 (0·01–037)	0·32 (0·06–1·62)	0·10 (0·02–0·45)	0·15 (0·03–0·75)
	*P* value		0·00		0·001	0·17	0·00	0·02
T4	OR (95% CI)	1	1·98 (1·24–3·14)	1	0·29 (0·14–0·63)	0·49 (0·23–1·07)	0·10 (0·04–0·22)	0·56 (0·25–1·28)
	*P* value		0·00		0·00	0·07	0·00	0·17
T10	OR (95% CI)	1	0·87 (0·54–143)	1	13·56 (6·12–30·03)	1·84 (0·81–4·20)	16·66 (7·21–38·53)	2·27 (0·97–5·29)
	*P* value		0·606		0·00	0·143	0·00	0·056
T15	OR (95%CI)	1	1·48 (0·72–3·05)	1	2·56 (0·75–8·77)	2·11 (0·66–6·67)	0·51 (0·20–1·30)	1·71 (0·53–5·50)
	*P* value		0·284		0·132	0·203	0·164	0·361

A, *Mannhaemia haemolytica* serotype A; MA, *Pasteurella multocida* serotype A; T, *Bibersteinia trehalosi* serotype T; OR, odds ratio, CI, confidence interval; Ref., reference category.

### RDA for evaluation of potential clustering and distribution of serotype mixed co-infections in sheep and goats

There was no single animal that was seronegative for all pathogens tested. Of 384 animals, 11·98%, 23·70%, 25·26%, 22·66% and 8·85% were seropositive to four, five, six, seven and eight different combinations of serotypes, respectively. That is, 355 (92·45%) animals were infected by four or more serotype combinations. A total of 103 different serotype combination patterns were observed, which are summarized in Table 4 and a detailed distribution listed in Table 5. The most prevalent co-positive pattern involved all serotypes except T10, and was found in 43/384 (11·2%) animals. Positivity to all eight serotypes was the next most frequent finding (34/384; 8·85%), and 30/384 (7·81%) were co-positive to A1A2T3T4T15. There were 56 patterns that were only observed in a single animal (Table 5).

**Table 4.** The distribution of total summarized combination patterns of mixed serotype co-infection per single animal among small ruminants (n = 384) of the studied areas in Tigray region

	No. of serotypes infecting single animal								
	1	2	3	4	5	6	7	8	Total
No. of various patterns for serotype combinations	3	6	13	25	28	20	7	1	103
No. of infected animal	4	6	19	46	91	97	87	34	384
% of infected animals	1·04	1·56	4·95	11·98	23·70	25·26	22·66	8·85	

Overall, eight serotypes were detected. No single negative animal was observed among all of the examined animals (*n* = 384) as each animal was infected by at least one or more serotypes. The eight serotypes displayed a total of 103 different (co)-infection patterns across the 384 infected animals using different serotype combinations. For example, 25·26% (97/384) of the animals were infected by six serotypes with 20 different combination (cluster) patterns.

**Table 5.** Distribution of the detailed serotype combination patterns (n = 103 patterns) for co-infection in small ruminants (n = 384) from five locations of Tigray region

Serotype combination pattern for co-infection	No. of infected animals of 384 examined	% of infected animals of 384	No. of serotypes involved in the pattern, out of 8	Serotype combination pattern for co-infection	No. of infected animals of 384 examined	% of infected animals of 384	No. of serotypes involved in the pattern, out of 8
A1A2A7MAT3T4T15	43	11·20	7	A1A2T3T4T10T15	1	0·26	6
A1A2A7MAT3T4T10T15	34	8·85	8	A1A2A7MAT4T10	1	0·26	6
A1A2T3T4T15	30	7·81	5	A1A2A7MAT3T4	1	0·26	6
A1A2A7T3T4T15	22	5·73	6	A2PAT3T4T15	1	0·26	5
A1A2A7MAT3T10T15	19	4·95	7	A2A7T4T10T15	1	0·26	5
A1A2MAT3T4T15	13	3·39	6	A2A7MAT3T4	1	0·26	5
A1A2A7MAT10T15	12	3·13	6	A2A7MAT3T15	1	0·26	5
A1A2MAT3T4T10T15	10	2·60	7	A2A7MAT3T10	1	0·26	5
A1MAT3T4T15	9	2·34	5	A1A7T3T4T10	1	0·26	5
A1A7MAT3T4T15	8	2·08	6	A1A7MAT4T15	1	0·26	5
A1A7MAT3T4T10T15	6	1·56	7	A1A7MAT4T10	1	0·26	5
A1A2A7MAT3T15	6	1·56	6	A1A7MAT3T15	1	0·26	5
A2A7MAT3T4T15	5	1·30	6	A1A2MAT3T10	1	0·26	5
A2A7MAT3T10T15	5	1·30	6	A1A2MAT3T10	1	0·26	5
A1A7T3T4T15	5	1·30	5	A1A2A7T3T4	1	0·26	5
A1A2MAT3T15	5	1·30	5	A1A2A7T3T4	1	0·26	5
A1MAT3T15	5	1·30	4	A1A2A7T10T15	1	0·26	5
A1A2T3T15	5	1·30	4	A1A2A7MAT3	1	0·26	5
A2A7MAT3T4T10T15	4	1·04	7	T3T4T10T15	1	0·26	4
A1A2A7MAT4T10T15	4	1·04	7	PAT3T4T15	1	0·26	4
A1A2MAT3T10T15	4	1·04	6	A7MAT3T15	1	0·26	4
A1A2A7MAT3T10	4	1·04	6	A7MAT10T15	1	0·26	4
A7MAT3T10T15	4	1·04	5	A2MAT3T4	1	0·26	4
A2A7T3T4T15	4	1·04	5	A2MAT3T15	1	0·26	4
A1A7MAT10T15	4	1·04	5	A2MAT10T15	1	0·26	4
A7MAT4T15	4	1·04	4	A2A7MAT10	1	0·26	4
A1T3T15	4	1·04	3	A1A7T4T15	1	0·26	4
A1A7MAT4T10T15	3	0·78	6	A1A7T3T15	1	0·26	4
A1A7MAT3T10T15	3	0·78	6	A1A7T3T10	1	0·26	4
A1A2A7T3T10T15	3	0·78	6	A1A7MAT10	1	0·26	4
A2MAT3T10T15	3	0·78	5	A1A2T3T10	1	0·26	4
A1MAT3T10T15	3	0·78	5	A1A2MAT3	1	0·26	4
A1A2A7T3T15	3	0·78	5	A1A2A7MA	1	0·26	4
A7MAT10T15	3	0·78	4	T3T4T15	1	0·26	3
A2T3T4T15	3	0·78	4	T3T4T15	1	0·26	3
A1T3T4T15	3	0·78	4	MAT3T15	1	0·26	3
A7MAT15	3	0·78	3	A7T3T15	1	0·26	3
A7MAT3T4T10T15	2	0·52	6	A7MAT3	1	0·26	3
A2A7MAT10T15	2	0·52	5	A2T4T15	1	0·26	3
A1A7T4T10T15	2	0·52	5	A1MAT3	1	0·26	3
A1A2T3T10T15	2	0·52	5	A1A7MA	1	0·26	3
A2A7T3T15	2	0·52	4	A1A2T3	1	0·26	3
A1A7MAT15	2	0·52	4	A1A2MA	1	0·26	3
A1A2T3T4	2	0·52	4	T3T4	1	0·26	2
A1A2T3T4	2	0·52	4	T3T15	1	0·26	2
A2T3T4	2	0·52	3	A7T15	1	0·26	2
A7	2	0·52	1	A7T10	1	0·26	2
A1A2A7T3T4T10T15	1	0·26	7	A2T3	1	0·26	2
A2A7T3T4T10T15	1	0·26	6	A2T15	1	0·26	2
A2A7MAT4T10T15	1	0·26	6	T4	1	0·26	1
A1MAT3T4T10T15	1	0·26	6	T3	1	0·26	1
A1A7T3T4T10T15	1	0·26	6				

**Fig. 1.**
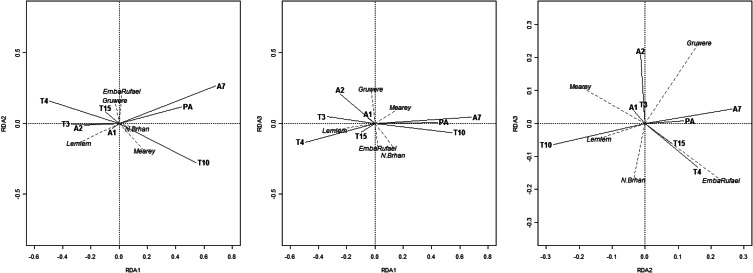
*Mannheimia, Pasteurella* and *Bibersteinia* serotype co-infections analysed for potential of clustering in sheep and goats in different peasant associations (PAs). Triplots of the three significant redundancy analysis (RDA) axes, showing relationships between explanatory and response variables. Solid lines represent serological responses, dashed lines represent PAs. Angles between variables represent their correlations.

Subsequently, the level of within-animal clustering (co-positivity) of serotypes and risk factors affecting clustering tendencies were evaluated using RDA (Fig. 1). Only PA explained a significant proportion of the variation (adjusted *R*^2^ = 0·16) in the serological responses. After this risk factor was accounted for, *B. trehalosi* serotypes T3 and T4 were observed to be positively correlated within animals; however, T4 serotype was observed to have negative correlations with the T10 strain. *M. haemolytica* strain A7 was clustered with *P. multocida* serotype A, and to a lesser extent with *B. trehalosi* serotype T10 within animals.

## DISCUSSION

Pasteurellosis is a pneumonic or acute systemic disease of small ruminants, with the precise syndrome determined by specific serotypes. In this regard pasteurellosis is a significant problem in extensive free-roaming smallholder farming conditions as animals are exposed to a wide spectrum of stressful conditions [29, 30]. Serotyping remains an important step in delineating *P. multocida*, *B. trehalosi* and *M. haemolytica* isolates, as antigens for vaccines are still based on capsular types and cross-reaction is absent in different serotypes [4]. In this study, the abundance, distribution, risk factors for positivity to different serotypes, and potential within-animal clustering of seropositivity were assessed in *Pasteurella, Mannheimia* and *Bibersteinia* in sheep and goats in selected areas of Tigray region.

Overall, 98·7% prevalence was found for both *M. haemolytica* and *B. trehalosi* (any serotype) and 66·9% for *P. multocida* serotype A in this study, indicating the former two are particularly widespread. At the individual serotype level, the prevalence was highest for the T15, T3 and A1 serotypes. A previous report from Northern Ireland similarly reported that *B. trehalosi* was more common than *M. haemolytica* in sheep [31]. However, other studies have reported that *M. haemolytica* is the most frequent serotype in sheep and goats in Egypt [32] and in northwestern Ethiopia [30]. In the current study, all sampled animals were seropositive to at least one serotype. This could be due to the commensal presence of some of these organisms as normal constituents of the nasal and pharyngeal microflora of healthy ruminants [33–35]. Healthy small ruminants are frequent carriers of these commensal opportunistic bacteria genera which tend to impose significant health and economic problems mainly when animals are under stressful conditions [34, 35]. However, the high prevalence and multiple circulating serotypes in the area could cause substantial animal health and economic impacts, as the sub-optimal animal management by smallholder farmers would exacerbate the impact of pasteurellosis.

The current study evaluated 384 sera for eight serotypes using the IHA test. All eight tested serotypes were frequently identified among sheep and goats: A1 (79·4%), A2 (73·7%), A7 (65·5%), MA (66·9%), T3 (84·4%), T4 (62·5%), T10 (41·4%) and T15 (89·6%). A complication of pasteurellosis control is that known serotypes do not produce cross-immunity [4]. Complex clonal diversity of strains has been observed within *M. haemolytica* serotypes [36] particularly within A1 and A2 [37] and within *B. trehalosi* T4 and T15 serotypes [38] in which each unique clone appears to have host-specific tropism [9] and can act as a primary pathogen, causing disease outbreaks in the specific host species. In this study the most common serotypes in goats were serotypes A1, A2, T3 and T4 and in sheep A7 and T10. Even after controlling for the variation between different PAs, goats had higher odds of T3 and T4 infection compared to sheep. In a similar study in Hungary, only serotype A2 was frequently detected in goats, and the remaining serotypes were very rare, while all serotypes were detected in sheep except A14 [39]. Variation in the detection of serotypes between sheep and goats could be explained by differing host tropism between serotypes [9]. In this study *P. multocida* serotype A was another important infection which was detected in sheep with prevalence slightly higher than in goats. *P. multocida* is emerging as a common pneumonic pasteurellosis disease in sheep and goats [40, 41], it is more commonly encountered in tropical and subtropical regions [41].

Variations in the age, sex and body condition of the animals did not appear to impact the seroprevalence of the majority of the studied serotypes. However, serotypes A1 and T15 were more prevalent in adult age (>2·5 years) and in slightly heavier bodyweight (11–20 kg) small ruminants, respectively. It is not known if serotypes A1 and T15 have unique tropism to animals of specific age groups or bodyweight, although host-specific tropism has been reported for a number of serotypes and for subgroups within a serotype [9, 36, 38, 42, 43].

One limitation of serosurveys is their inability to differentiate past exposure from recent infection. Thus, the current study could not discriminate whether the higher seroprevalence in the adult age groups was a reflection of the long-term cumulative accumulation of antibodies due to past exposure or due to recent or current infection. Generally, however, *M. haemolytica* causes septicaemia in young lambs and pneumonia in all ages while *B. trehalosi* causes acute systemic disease affecting principally the upper alimentary tract and lungs in young adults [44].

Each locality (PA) had a different magnitude of prevalence for each of the eight serotypes studied and only PA explained a significant proportion of the variation (adjusted *R*^2^ = 0·16) in the serological responses. Variation in seroprevalence between PAs may be due to geographical variation in type and level of predisposing factors, serotype abundance and/or the immune status of the studied animals [29–32]. While the most prevalent *Pasteurella* strain varied between PAs, even within PAs, there was considerable variation in the serotypes to which each animal had been exposed, with the two major clusters appearing to be A7, MA and T10; or T3 and T4. The distribution of these five serotypes through the population of the studied small ruminants of different locations (PAs) points towards contagious spread while the remaining three serotypes seem a totally endogenous infection with limited evidence of spread within the wider flock. Similar findings have been reported previously [42, 43].

The clustering of certain serotypes of *Pasteurella* within animals may reflect common pathways of exposure or differing genetic or acquired susceptibility in certain groups of animals. While not significant in RDA, multivariable logistic regression suggested that T3 and T4 were more likely to be found in goats than sheep; however, the results from the RDA suggest that, even in sheep, these strains are more likely to be found together than would be expected if the two strains were independent. After the effect of PA was accounted for, *B. trehalosi* strains T3 and T4 were observed to be positively correlated within animals: however, T4 strain was observed to have negative correlations with the T10 strain, suggesting they were less likely to be found within the same animal than might be expected under an assumption of independence. *M. haemolytica* strain A7 was clustered with *P. multocida* serotype A, and to a lesser extent with *B. trehalosi* strain T10 within animals.

The implications for vaccine production of these findings are that, due to the considerable variation in strain exposure both between PAs and between animals in the same PA, it is difficult to select serotypes for a polyvalent vaccine based on serology alone. However, as many *Pasteurella* infections may be clinically silent, or only be significant as secondary infections in the presence of other pathogens, combination of this serological data with data from clinical case presentations and pathogen isolation may provide valuable information in selecting the serotypes of greatest importance in this population. Importantly, the significant variation between PAs may suggest caution should be exercised when extending these findings to other areas.

## CONCLUSION

The current serological study revealed the widespread distribution of eight serotypes of pasteurellosis agents among localities, sheep and goats in Tigray region. Of the risk factors evaluated, age (serotype A1) and bodyweight (serotype T15) were significantly associated with infection, but sex was not significant. Serotypes A1, A2, T3 and T4 were significantly associated with goats; A7 and T10 with sheep; *P. multocida* serotypes A and T15 with both sheep and goats; A1 and T15 with all the five PAs while serotypes A2, A7, T3, T4, and T10 were associated with at least one of the five PAs. Only PA explained a significant proportion of the variation (adjusted *R*^2^ = 0·16) in the serological responses. After the effect of PA was accounted for, T3 and T4 were positively correlated with co-infection while T4 and T10 had negative correlations and were less likely to be found within the same animal. Serotype A7 was clustered with *P. multocida* serotype A, and to a lesser extent T10 within animals. Overall 355 (92·45%) animals were infected with four or more serotype combinations with potential of clustering serotype per animal. The diversity of serotypes observed here indicates that currently available vaccines are likely to be unable adequately to protect against pasteurellosis. The results of this study clearly indicate the need for further investigation to better understand the epidemiology of pasteurellosis across a wider geographical area, including investigation of the clonal diversity of pasteurellosis pathogens responsible for clinical cases, in order to inform recommendations for the selection of serotypes for inclusion in polyvalent vaccines.
